# Epidemiological characteristics of *P. vivax* asymptomatic infections in the Peruvian Amazon

**DOI:** 10.3389/fcimb.2022.901423

**Published:** 2022-08-31

**Authors:** Elizabeth Villasis, Stefano S. Garcia Castillo, Mitchel Guzman, Julian Torres, Joaquin Gomez, Katherine Garro, Ana Maria Cordova, Carolina Reategui, Caroline Abanto, Joseph Vinetz, Dionicia Gamboa, Katherine Torres

**Affiliations:** ^1^Laboratorio de Malaria, Laboratorios de Investigación y Desarrollo, Facultad de Ciencias y Filosofía, Universidad Peruana Cayetano Heredia, Lima, Peru; ^2^Instituto de Medicina Tropical Alexander von Humboldt, Universidad Peruana Cayetano Heredia, Lima, Peru; ^3^Laboratorio de Malaria: Parásitos y Vectores, Laboratorios de Investigación y Desarrollo, Facultad de Ciencias y Filosofía, Universidad Peruana Cayetano Heredia, Lima, Peru; ^4^Laboratorio ICEMR Amazonia y Enfermedades Emergentes, Universidad Peruana Cayetano Heredia, Iquitos, Peru; ^5^Laboratorio ICEMR−Amazonia y Enfermedades Infecciosas Emergentes, Laboratorios de Investigación y Desarrollo, Facultad de Ciencias y Filosofía, Universidad Peruana Cayetano Heredia, Lima, Peru; ^6^Section of Infectious Diseases, Department of Internal Medicine, Yale School of Medicine, New Haven, CT, United States; ^7^Departamento de Ciencias Celulares y Moleculares, Facultad de Ciencias y Filosofía, Universidad Peruana Cayetano Heredia, Lima, Peru

**Keywords:** asymptomatic, infection, *P. vivax*, parasitemia, biochemical, hematological, parameters

## Abstract

**Introduction:**

Herein, we tested the hypothesis that Asymptomatic *P. vivax* (Pv) infected individuals (Asym) feature different epidemiological, clinical and biochemical characteristics, as well as hematological parameters, potentially predictive of clinical immunity in comparison to symptomatic Pv infected individuals (Sym).

**Methodology:**

Between 2018 - 2021, we conducted 11 population screenings (PS, Day 0 (D0)) in 13 different riverine communities around Iquitos city, in the Peruvian Amazon, to identify Pv Sym and Asym individuals. A group of these individuals agreed to participate in a nested case - control study to evaluate biochemical and hematological parameters. Pv Asym individuals did not present common malaria symptoms (fever, headache, and chills), had a positive/negative microscopy result, a positive qPCR result, reported no history of antimalarial treatment during the last month, and were followed-up weekly until Day 21 (D21). Control individuals, had a negative malaria microscopy and qPCR result, no history of antimalarial treatment or malaria infections during the last three years, and no history of comorbidities or chronic infections.

**Results:**

From the 2159 individuals screened during PS, data revealed a low but heterogeneous Pv prevalence across the communities (11.4%), where most infections were Asym (66.7%) and submicroscopic (82.9%). A total of 29 Asym, 49 Sym, and 30 control individuals participated in the nested case - control study (n=78). Ten of the individuals that were initially Asym at D0, experienced malaria symptoms during follow up and therefore, were included in the Sym group. 29 individuals remained Asym throughout all follow-ups. High levels of eosinophils were found in Asym individuals in comparison to Sym and controls.

**Conclusion:**

For the first-time, key epidemiological, hematological, and biochemical features are reported from Pv Asym infections from the Peruvian Amazon. These results should be considered for the design and reshaping of malaria control measures as the country moves toward malaria elimination.

## Introduction

*Plasmodium vivax* (Pv) is the most geographically widespread human malaria parasite. However, compared to *P. falciparum* (Pf), global malaria efforts have been less successful at reducing the burden of Pv, owing to its unique biology and treatment complexity (World Health Organization, 2021). The key biological features of Pv which contribute to its ability to circumvent existing malaria control tools include: frequent low-density parasite prevalence, which is mostly only detectable by molecular tools and not by standard methodologies such as microscopy and Rapid Diagnostics Tests, conventionally used by National Malaria Control Programs; and the presentation of persistent dormant liver stages (hypnozoites) as reservoirs, that are undetectable but reactivate to cause relapsing infections, difficult to predict. Pv infections also tend to reach peak gametocytemia before symptoms present themselves, making this species more transmissible than Pf. Although, Pv infection is thought to cause benign infection, the overall burden disproportionately affects poor people from remote rural regions, resulting in a considerable underestimated economic impact in society (Angrisano and Robinson, 2022).

The role of asymptomatic carriers in the natural history of malaria is not entirely understood (Almeida et al., 2021). Persistent, asymptomatic *Plasmodium* spp. infections carry health risks for the infected individual, including chronic anemia, increased maternal and neonatal mortality risks, impaired immune competence resulting in co-infections with invasive bacterial diseases, and cognitive impairment (Nguyen et al., 2018; Nyarko and Claessens, 2021). Whether this type of infection is a reservoir and, therefore, important for transmission, is under debate. While some studies have shown that asymptomatic and submicroscopic infections contribute to transmission, others have shown very limited infectivity in mosquitoes (Edwards et al., 2021; Ferreira et al., 2022; Moreno et al., 2022). However, asymptomatic carriers may cause imported malaria, and transfusion or organ-transplantation malaria (Sato, 2021). Thanks to economic development and transportation we now live in a more globalized world in which global travel is much easier. Therefore, there is now an urgent need to control asymptomatic carriers of malaria parasites.

Recent results from large cohort studies in Peru carried out by the Amazonian Center of Excellence in Malaria Research (ICEMR), highlight the persistence and high prevalence of Pv asymptomatic malaria infections as detected by molecular tools (Rosas-Aguirre et al., 2015a; Carrasco-Escobar et al., 2017a; Moreno-Gutierrez et al., 2018; Moreno et al., 2022). Therefore, Active Case Detection and treatment of individuals with submicroscopic asymptomatic parasitemia will be a critical step to eliminate malaria in Peru, as part of the “Malaria elimination Plan” (Ministerio de Salud del Peru (MINSA), 2022). To achieve the latter, the use of highly sensitive and specific molecular detection tools, along with serological markers and geolocalization instruments will be critical to identify malaria asymptomatic hotspots and associated risk factors (Rosas-Aguirre et al., 2015b).

To better characterize asymptomatic infections in the Peruvian Amazon and to extend our understanding of its biological process, we carried out eleven cross-sectional studies in a total of thirteen communities from the Peruvian Amazon between 2018 and 2021 to search for malaria cases. Next, we carried out a nested case - control study between Pv symptomatic (Sym), asymptomatic (Asym), and control healthy individuals. We tested the hypothesis that Pv Asym infected individuals feature different epidemiological, clinical, and biochemical characteristics, and hematological parameters predictive of clinical immunity compared to Pv Sym infected individuals.

## Materials and methods

### Study area

**Figure 1:**
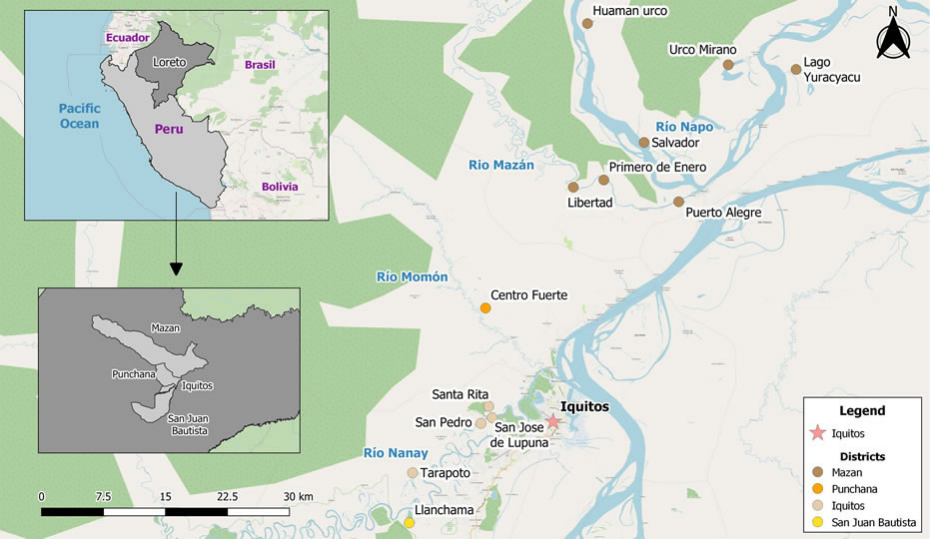
Study areas in Loreto region, Peruvian Amazon. Communities from four districts were included: Llanchama in the San Juan Bautista district (yellow dot); Tarapoto, Santa Rita, San José de Lupuna, and San Pedro in the Iquitos district (bisque dots); Centro Fuerte in the Punchana district (orange dot); Libertad, Primero de Enero, Puerto Alegre, Huaman Urco, Salvador Urco Miraño, and Lago Yuracyacu in the Mazan district (brown dots).

Eleven population screenings (PS) or cross-sectional studies were conducted between September 2018 and November 2021 in a total of thirteen riverine communities, six of which are located near to Iquitos City: Tarapoto (-73.4070° W, -3.8046° S), Santa Rita (3.7321° S, 73.3241° W), San José de Lupuna (3.745° S, 73.323° W), Llanchama (3.8591° S, 73.4106° W), and San Pedro (3.7508° S, 73.3324° W) located at the basin of the Nanay river and Centro Fuerte community (3.6251° S, 73.3275° W) located at the Momon river; and seven communities from the Mazan district: Libertad (3.496° S, 73.234° W), Primero de Enero (3.479° S, 73.199° W) and Puerto Alegre (3.510° S, 73.116° W) located at the basin of the Mazan river; and Huaman Urco (-73.2164° W, -3.3150° S), Salvador (-73.1547° W, -3.4446° S), Urco Miraño (3.361° S, 73.064° W) and Lago Yuracyacu (3.365° S, 72.989° W) located at the basin of the Napo river (**Figure 1**, **Supplementary Tables 1–3**.). The selection of the collection sites fulfilled two main criteria: 1) high positivity malaria index in the previous eight weeks before population screening this data was collected from Weekly Notification Office of Epidemiology of the Regional Direction of Health of Loreto (DIRESA) and 2) easy access for individuals follow-up and subsequent enrollment.

### Cross-sectional study

During the PS, all individuals (>18 years old) that agreed to participate in the study, after giving informed consent, filled out a comprehensive survey related to malaria history and symptoms, and were asked for finger-prick blood samples for malaria diagnosis by microscopy and qPCR (the day of the PS was also called Day 0, D0). To prevent bias, the individuals were not given a symptoms list; instead, a self-explanation from each one’s symptoms was recorded. Microscopy and molecular diagnosis were performed within 24 hours after sample collection. Pv Sym individuals were classified as people presenting at least one malaria symptom (fever, headache, and chills), a Pv positive or negative microscopy result, and a Pv positive qPCR result at the time of PS (D0). Pv Asym individuals did not present malaria symptoms during the previous seven days before PS, had a Pv positive or negative microscopy result, and a Pv positive qPCR result at the time of PS (D0). Sym individuals detected with *Plasmodium* spp., infection by microscopy or qPCR, received immediate appropriate treatment at the health centers, following the Peruvian Ministry of Health Guidelines after sample collection (Ministerio de Salud del Peru, 2015). We obtained microscopy and qPCR results from a total of 2159 individuals.

### Samples collection

Finger-prick blood samples were collected for thick and thin blood smear for microscopy analysis. During the first four PS’, filter papers impregnated with whole blood were used for qPCR analysis (Rosas-Aguirre et al., 2020); starting the fifth PS, whole blood samples were collected from finger-prick using microtainers with EDTA for qPCR (Vacutest, Kima ^®^, Padua, Italy). Whole blood samples in microtainers were centrifuged to separate red blood cells from plasma, and stored at −20°C until processing (Rosas-Aguirre et al., 2020). qPCR was performed within 24 hours after sample collection (Moreno et al., 2022).

### Nested case - control study

The participants from PS with a positive Pv qPCR and/or microscopy result were invited to participate in the nested case - control study if they met the inclusion criteria. The overall exclusion criteria were being pregnant, diagnosis of mixed infection with *Plasmodium* spp. parasites by qPCR or microscopy, and oral report of history of common chronic or infectious diseases such as diabetes, HIV, or being immunosuppressed. Pv Asym individuals did not present malaria symptoms during the previous seven days before PS, had a Pv positive or negative microscopy result, and a Pv positive qPCR result at the time of PS (D0) or any of the follow-up days (D7, D14, D21), and reported no history of antimalarial treatment during the last month (Torres et al., 2014). Pv Sym individuals were classified as people presenting at least one malaria symptom (fever, headache, and chills), a Pv positive or negative microscopy result, and a Pv positive qPCR result at the time of PS (D0) or any of the follow-up days (Lisbôa et al., 2022). Healthy controls were individuals with residency in Iquitos city, had no history of antimalarial treatment or malaria infections during the last three years, had negative results for *Plasmodium* spp. infection by microscopy and qPCR, and reported no oral history of comorbidities or chronic infections. On the last day of follow-up, Pv Sym (n = 49), Pv Asym (n = 29), and control individuals (n = 30), were invited to donate 60 – 100 mL of blood for immunological, biochemical, and hematological testing in our laboratory facilities in Iquitos city. A complete medical evaluation was carried out by a physician prior to sample collection. Sym individuals detected with *Plasmodium* spp., infection by microscopy or qPCR at D0 or any day during follow-up, received immediate appropriate treatment at the health centers, following the Peruvian Ministry of Health Guidelines, after sample collection. All the Asym individuals received counselling and were directed to seek healthcare in a malaria reference center in case symptoms appeared during the follow-up period, samples were collected before individuals received antimalaria treatment. After sample collection, all the participants that had a positive malaria screening by qPCR were referred to the nearest health center for antimalaria treatment following the Peruvian Ministry of Health Guidelines. Pv non complicated malaria in adults were treated with chloroquine 10mg/Kg on days 1 and 2, and 5mg/Kg on day 3, plus primaquine 0.5 mg/Kg/day for seven days; while, Pf non complicated malaria in adults was treated with mefloquine 12.5mg/Kg/day for 2 days, plus artesunate 4 mg/kg/day for 3 days, plus primaquine 0.75mg/Kg/day for 1 day (Ministerio de Salud del Peru, 2015).As a parallel strategy to reach our sample size in a shorter time period, Pv Sym individuals were also enrolled by passive case detection at different health centers in Iquitos city (n = 10/49), these individuals also received immediate appropriate treatment at the health centers, following the Peruvian Ministry of Health Guidelines, after sample collection.

### Laboratory procedures

#### Light microscopy

Thick and thin smears were stained for 10 min with a 10% Giemsa solution, and parasite density was calculated after counting the number of parasites for 200 white blood cells (WBCs) in the thick smear, assuming a concentration of 8000 WBCs/μL. Slides were read on-site and then again by a microscopy expert in our laboratory at Iquitos city. A slide was declared negative if no malaria parasites were found after examining 200 fields (MINSA, Instituto Nacional de Salud et al., 2003). Quality control was performed blindly on all positive slides and 10% of randomly chosen negative slides by an expert microscopist from the Referential Laboratory of the Loreto Regional Health Direction (DIRESA). Discordant results were reassessed by a second senior expert microscopist.

#### Real-time quantitative PCR

The E.Z.N.A.^®^ Blood DNA Mini Kit (Omega Bio-tek^®^, Georgia, USA) was used to extract genomic DNA from 40 μL of red blood cells with EDTA or 0.72 cm^2^ of filter paper impregnated with whole blood following the manufacturer’s instructions with slight modifications (i.e., addition of TEN buffer (20 mM Tris-HCl, pH 8.0; 2 mM EDTA, pH 8.0; 0.2 M NaCl) supplemented with SDS 10% w/v). Extracted DNA was stored at 4°C for immediate use and at −20°C for later analyses.

*Plasmodium* spp. screening was performed using a qPCR that targets the cytochrome oxidase III (COX-III) mitochondrial gene (216 bp) for the detection *of Plasmodium spp* DNA using SYBR Green. Briefly, 5 μL of extracted DNA was mixed with 0.2 μM of primers. COXIIIF: 5’-CGGTAGATAGGGAACAAACT-3’ and COXIIIR: 5’-CTTTGCCTGGAGGTTACG-3’, 12.5 μL PerfeCTa^®^ SYBR^®^ Green FastMix^®^ and 6.5μL PCR-grade water per reaction. Reactions were carried out in an Real-Time PCR CFX96TM Bio-Rad (Bio-Rad, USA) using the following qPCR conditions: initial denaturation at 95°C for 3 min, followed by amplification for 45 cycles of 15 s at 95°C, 15 s at 60°C, and 15 s at 68°C, and a final extension of 3 min at 70°C. Amplification was immediately followed by a melting program consisting of a stepwise temperature increase from 70°C to 90°C with an increase of 0.5°C/s up for a total of 5 s. Positive, negative, and blank controls were added to each plate. The same positive control was added to each plate to ensure inter-assay comparability. Analysis of the differences in melting curves provided an accurate differentiation between positive and negative samples.

Positive samples *Plasmodium spp* identified during screening were used to detect the small rRNA subunit (18S) of Pv and Pf species using previously described probes with small modifications (Rougemont et al., 2004; Pincelli et al., 2018). Briefly, 5 μL of extracted DNA was mixed with 0.1 μM of primers 5’- GTTAAGGGAGTGAAGACGATCAGA – 3’ and 5’ – AACCCAAAGACTTTGATTTCTCATAA – 3’. Moreover, the following species-specific Taqman probes were added in separated reactions: Pv 5’ VIC – AGCAATCTAAGAATAAACTCCGAAGAGAAAATTCT- QSY 3’ and Pf 5’ FAM – AGCAATCTAAAAGTCACCTCGAAAGATGACT – QSY 3’ at a concentration of 0.08 µM. Finally, 10 μL of TaqMan™ Environmental Master Mix 2.0 (Applied Biosystems), and 4.44 μL PCR-grade water were added per reaction. Reactions were performed in a Real-Time PCR CFX96TM Bio-Rad (Bio-Rad, USA) using the following qPCR conditions: initial denaturation at 95°C for 10 min and amplification for 45 cycles of 15 s at 95°C, 60 s at 60°C. Serial dilutions of positive controls of Pf and Pv DNA at five concentrations between 20 – 2 par/μL were prepared and a standard curve was used to calculate the number of par/μL in a sample (Moreno et al., 2022).

#### Clinical laboratory

We measured the following hematological parameters: leukocytes, red blood cells (RBC), hemoglobin, hematocrit, platelets, mean corpuscular volume (MCV), mean corpuscular hemoglobin (MCH), mean corpuscular hemoglobin concentration (MCHC), neutrophils, lymphocytes, monocytes, eosinophils, and basophils. We also evaluated the following biochemical clinical analytes: alanine transaminase (ALT), aspartate aminotransferase (AST), urea, creatinine, alkaline phosphatase (ALP), bilirubin, direct bilirubin, indirect bilirubin, gamma-glutamyl transpeptidase, glucose, proteins, potassium, and sodium. These parameters were measured for all enrolled individuals in the nested case -control study. The automated Pentra 60C Hematology Analyzer (Horiba, Kyoto, Japan) and Cobas c311 Automated Analyzer (Roche Laboratories, Basel, Switzerland), were used for hematological and biochemical assays, respectively. All the 26 analytes were measured using recommended commercial kits from the same brand of the Automated Analyzers. International references values were applied for assessment. For all procedures, the standards of laboratory quality control were followed.

#### Data analysis

Data were analyzed using R statistical software (Version 4.0.0) and RStudio software (Version 1.2.5042) and models packages “rstatix”, “stats”, “epiR” and “UpSetR”. For all analyses, p values < 0.05 were considered statistically significant. For the cross-sectional study, a Fisher’s exact test was used to evaluate the differences in the proportion of malaria cases between PS in communities with more than one PS. Two independent univariable logistic regression analysis was performed to determine associated factors to develop an Asym malaria infection. The first analysis included sociodemographic information such season, sex, age group, diagnostic status, travel history and occupation. The second analysis was focused on the household characteristics. The multivariable logistic regression analysis only included factors from the univariable analysis with results of likelihood ratio test (p-values <0.1). The two final predictive models contained all potential variables that were significantly associated with Asym malaria infection. For the nested case-control study, a Kruskal-Wallis test was used to compare results between clinical status groups (Asym, Sym, and controls), and a Dunn test as *post-hoc* when necessary.

Geographical analysis was done using QGis (v. 3.10.10) and R packages’ ‘sp’, ‘spdep’, and ‘sf’. First, Heatmaps were constructed with the number of Pv Sym and Pv Asym malaria cases confirmed by qPCR per house in each community employing a kernel density estimation (KDE) on QGis. Later, two indexes were calculated to assess spatial autocorrelation of the number of Asym cases in each site: Global Moran’s I and Local Moran’s I. If a village had more than one visit, indexes were calculated for each one of those. As our dataset was composed of coordinates, we defined neighbors by the distance between them. To avoid isolates, we calculated the threshold distance at which any point that had at least one neighbor inside its own village. From here, we performed 99,999 Markov chain Monte Carlo (MCMC) permutations to assess the significance of our Global Moran’s Index. Finally, to assess local spatial autocorrelation, we computed the Local Moran’s I using the same minimal distance for each village and correcting for false discoveries (FDR). This process allows us to classify houses in four categories: High - High, houses with a high number of Asym cases surrounded by points with a high number of them; Low - Low, houses with a low number of Asym cases surrounded by houses with a low number of them, and High - Low and Low - High respectively.

#### Ethics

The studies involving human participants were reviewed and approved by the Ethical Committee of the Universidad Peruana Cayetano Heredia (Lima, Peru). The participants provided written informed consent at the beginning of the study during PS (SIDISI code 101518 approved on 10/27/2017) and to participate in the nested case - control study (SIDISI code 101497 approved on 10/26/2017).

## Results

### Population socio−demographic and household characteristics

A total of 2159 individuals (≥18 years old) and 688 households were surveyed across 11 cross-sectional studies carried out between 2018 and 2021, in 13 communities in four districts, with different living conditions from the Peruvian Amazon (**Supplementary Tables 1, 2**). Overall, 50.7% (1094/2159) were female, and individuals had a median age of 43 years old (IQR = 31 – 57 years old). Of these, 50.4% (1088/2159) had primary education, ranging between 33.8% and 71.7% across the communities, followed by secondary education (34.0%, 734/2159). More than half of participants (57.2%, 1236/2159) reported working on outdoor activities, such as logging, fishing, or farming. Interestingly, most participants did not report travel history outside their communities in the previous month of the PS (66.9%, 1444/2159) (**Supplementary Table 1**).

More than half of the screened households were completely closed (62.9%, 433/688), had at least one bedroom (52.6%, 362/688), and no more than two beds (53.1%, 365/688) per house. The predominant material for walls and floors was wood 91.9% (632/688) and 57.6% (396/688), respectively; and roofs were mainly made of calamine or tin (69.6%, 479/688). The availability of potable water was scarce in most communities (4.1%, 28/688), and the primary water source for drinking was obtained from adjunct rivers or rain (53.1%, 365/688). The proportion of households that possessed more than one net treated with insecticide was high (80.4%, 553/688), and a low proportion of residents (34.0%, 234/688) reported their houses were sprayed with insecticide (**Supplementary Table 2**).

### *P. vivax* asymptomatic infections are predominant and missed by routine malaria diagnostics

**Figure 2:**
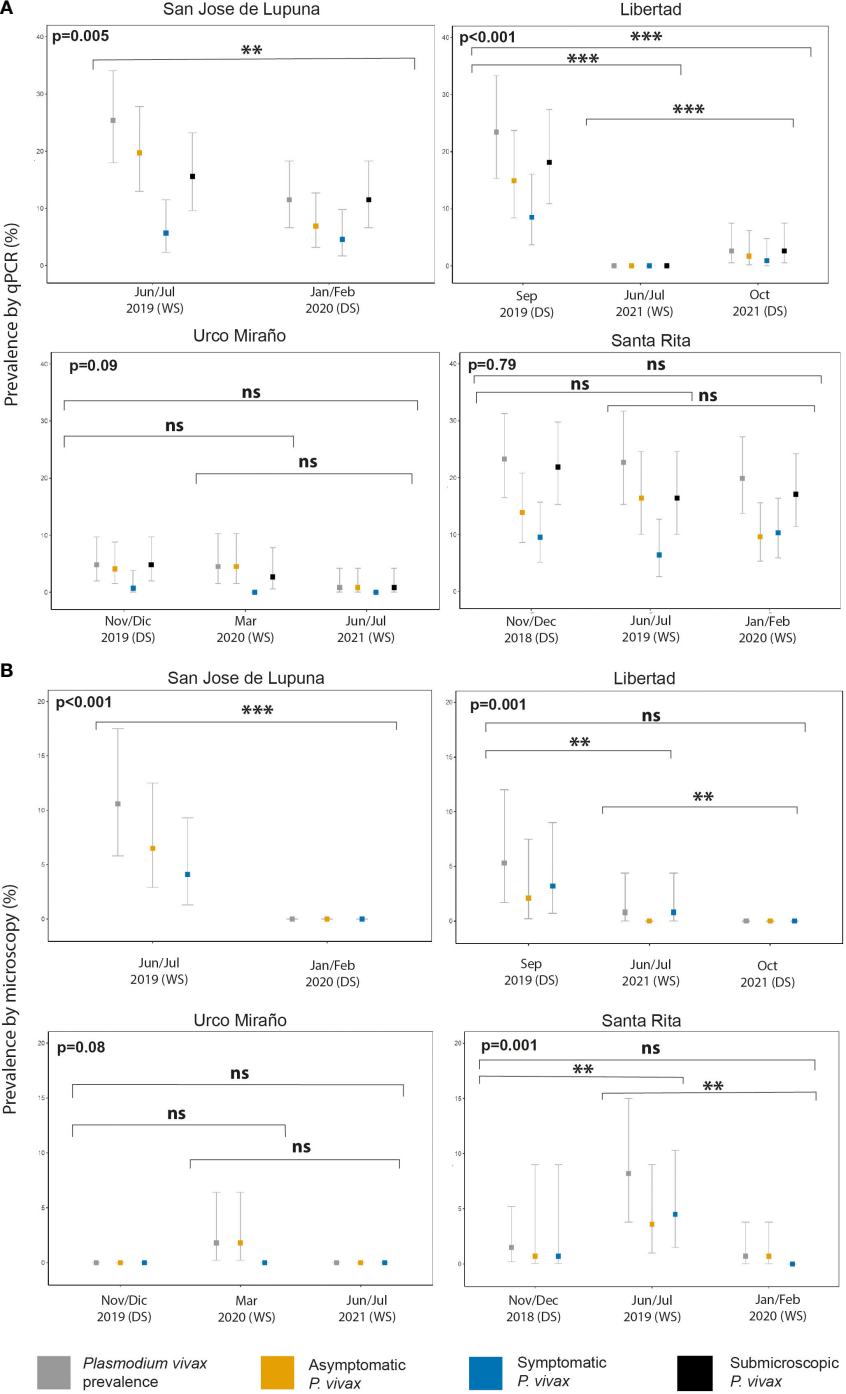
P*. vivax* malaria prevalence by population screening method [Microscopy **(A)** and qPCR **(B)**], and clinical status (asymptomatic, symptomatic, and submicroscopic infection) of communities with multiple population screenings throughout the study. WS, wet season; DS, dry season. **p < 0.01, ***p < 0.001. ns, no significant

Throughout the study, microscopy diagnosis underestimated Pv prevalence of infections by sixfold vs qPCR (1.9% vs 11.4%); therefore, qPCR was used as the reference method for confirmation of Pv infections for all analyses (**Figure 2**, **Supplementary Table S3**). Overall, two out of three malaria infections detected by qPCR were Asym (66.7%, 164/246). Pv Asym infections were mainly detected during the dry season 70.7% (116/164) when malaria prevalence is lower. Interestingly, most asymptomatic individuals were males 53.7% (88/164) above 48 years old (30.5%, 50/164) and had submicroscopic parasitemia 82.9% (136/164) (**Table 1**).

**Table 1:** Univariable and multivariable associated factor analysis of sociodemographic characteristics for *P. vivax* asymptomatic infection.** **

	Asymptomatic (n=164)		Symptomatic (n=82)		Univariable model			Multivariable model		
	n	%	n	%	OR	p value	95% CI	AOR	p value	95% CI
**Season**										
Dry	116	71.2	47	28.8	Ref.					
Wet	48	57.8	35	42.2	**0.56**	**0.037**	0.32 - 0.97	**0.55**	**0.0370**	0.32 - 0.96
**Sex**										
Female	76	64.4	42	35.6	Ref.					
Male	88	68.8	40	31.3	1.22	0.471	0.71 - 2.07			
**Age**										
18-28 years old	45	64.3	25	35.7	Ref.					
28-38 years old	33	64.7	18	35.3	1.02	0.962	0.48 - 2.18			
38-48 years old	36	69.2	16	30.8	1.25	0.568	0.58 - 2.72			
>48 years old	50	68.5	23	31.5	1.21	0.595	0.6 - 2.43			
**Diagnostic status**										
Microscopic	28	62.2	17	37.8						
Submicroscopic	136	67.7	65	32.3	1.27	0.485	0.64 - 2.47			
**Travel history last month**										
Negative	106	67.9	50	32.1	Ref.					
Positive	58	64.4	32	35.6	0.85	0.575	0.5 - 1.48			
**Outdoor Occupation (Logger, Fisher or Farmer)**										
Negative	71	61.7	44	38.3	Ref.					
Positive	93	71.0	38	29.0	1.52	0.125	0.89 - 2.59	1.53	0.1230	0.89 - 2.62

OR, odds ratio, CI, confidence interval. In bold, variables with significant values (*p < 0.05). Multivariable model: Null deviance: 245 on 313.2 degrees of freedom, Residual deviance: 243 on 306.5 degrees of freedom. AIC: 312.5.

San Jose de Lupuna proved to be the community with the highest prevalence of Pv infections, showing a prevalence of 25.4% (31/122) in Jun/Jul 2019; however, six months later (Jan/Feb 2020), this prevalence significantly decreased to 11.5% (15/130) (Fisher’s exact test, p = 0.005) despite being wet season (**Figure 2**). The proportion of Pv Asym infections detected by qPCR decreased (77.4% vs 60%) during the last PS, and the proportion of Pv Sym infections doubled (22.6% vs 40%) (**Supplementary Table 3**). Pv infection prevalence in Libertad community also showed a significant decrease trend from Sep 2019 to Jun/Jul 2021 to Oct 2021 (23.4% vs 0% vs 2.6%) (Fisher’s exact test, p < 0.001) (**Figure 2**), however the proportion of Pv Asym (63.6% vs 0% vs 66.7%) and Pv Sym infections (36.4% vs 0% vs 33.3%) detected by qPCR remained constant during the first and last PS (**Supplementary Table S3**). The same trend was observed in Urco Miraño community, from Nov/Dec 2019 to March 2020 to Jun/Jul 2021 (**Figure 2**), showing a non-significant decrease of Pv infection prevalence from 7% to 5% to 1% and a majority of Asym infections (85.7% vs 100% vs 100%), respectively (**Supplementary Table 3**).

Pv infection was barely reduced in Santa Rita community, where prevalence ranged from 23.3% (32/137) in Nov/Dec 2018 to 22.7% (25/110) in Jun/Jul 2019 and 19.9% (29/146) in Jan/Feb 2020 (Fisher’s exact test, p = 0.7902), reflecting the persistence of malaria transmission in this specific area in comparison to other communities (**Figure 2**). The proportion of Pv Sym and Asym infections was more dynamic in this community, ranging from 59.4% to 72% to 48.3% for Pv Asym and from 40.6% to 28% to 51.7% in the case of Pv Sym infections (**Supplementary Table 3**). Due to the selection criteria for study sites and the COVID-19 Pandemic restrictions, we could only carry out one PS for the other communities. Among these, Primero de Enero (100%), Huaman Urco (77.8%), and Llanchama (42.9%) presented the highest prevalence of Asym Pv infections (**Supplementary Table 3**). Overall, during this study, Pv infections were predominantly submicroscopic (82.9%, 201/246), asymptomatic (66.7%, 164/246) and more common in communities with low Pv prevalence (**Supplementary Table 3**).

The spatial analysis revealed Pv Asym hotspots with no clustering. We were able to identify more visual hotspots of Asym cases per house in communities like Santa Rita (PS2, PS4 and PS7, **Supplementary Figure 1A**), San Jose de Lupuna (PS4 and PS7, **Supplementary Figure 1B**), San Pedro (PS7, **Supplementary Figure 2**), and Libertad (PS5 and PS11, **Supplementary Figure 3**). Simultaneously, Urco Miraño, Tarapoto, Llanchama, Primero de Enero, Puerto Alegre, Huaman Urco, and Centro Fuerte showed an even distribution of these cases, with discrete points across the extension of the community (**Supplementary Figure 1C** and **Supplementary Figure 2**). Finally, Lago Yurac Yacu community did not present any Asym cases during the eighth PS (**Supplementary Figure 2**). In general, spatial autocorrelation analyses were non-significant at the global and local levels between hotspots in the study settings (**Supplementary Table S4**).

### Sociodemographic factors associated with *P. vivax* asymptomatic infection

First, a multivariable-adjusted analysis was carried out to determine the association of factors such as: season, sex, age, diagnostic status, travel history, and occupation, to Pv Asym infection (**Table 1**). We found that Asym infections were not associated to wet season (AOR = 0.56, 95% CI [0.32 – 0.96]). Neither sex, age, or travel history in the previous month were significantly associated with clinical status (**Table 1**). Additionally, we addressed the relevance of household characteristics to the presence of at least one Asym family member per house by performing a second multivariable-adjusted analysis (**Table 2**). Asym individuals were mainly found in houses with wooden walls (AOR=6.07, 95% CI [1.81 - 21.44]). Interestingly, even a mosquito net without insecticide exerted a protective effect on the houses as these families had 56% decrease odds to present an Asym family member (AOR= 0.44, 95% CI [0.20 - 0.94]. Significant results obtained using the variable of number of beds per house seem to lack biological plausibility and were more likely to be due to chance, as living in a house with more than three beds was not a factor associated to Asym infection (AOR = 0.36, 95% CI [0.16 - 1.05]) but living in a house with ≥ 4 beds had a trend associated to Asym infection although not significantly (AOR = 3.38, 95% CI [0.83 - 24.34]).

**Table 2:** Univariable and multivariable associated factor analysis of household characteristics for *P. vivax* asymptomatic infection.

	Asymptomatic individuals households (n=125)		Symptomatic individuals households (n=45)		Univariable model			Multivariable model		
	n	%	N	%	OR	p value	95% CI	AOR	p value	95% CI
**Mosquito net treated with insecticide**										
Negative	21	65.6	11	34.4	Ref.					
Positive	104	75.4	34	24.6	1.6	0.263	0.68 - 3.61			
**Mosquito net without insecticide**										
Negative	65	80.2	16	19.8	Ref.			Ref.		
Positive	60	67.4	29	32.6	0.51	0.060	0.25 - 1.02	**0.44**	**0.039**	**0.20 - 0.94**
**Number of bedrooms**										
0 - 1 bedrooms	45	66.7	22	33.3	Ref.					
2 bedrooms	41	76.5	13	23.5	1.54	0.292	0.70 - 3.52			
3 bedrooms	19	70.8	7	29.2	1.33	0.581	0.50 - 3.82			
>3 bedrooms	20	87.0	3	13.0	3.26	0.079	0.98 - 14.88			
**Number of beds**										
0 - 2 beds	54	74.0	19	26.0	Ref.			Ref.		
3 beds	31	62.0	19	38.0	0.57	0.160	0.26 - 1.25	**0.36**	**0.026**	**0.16 - 1.05**
4 beds	27	93.1	2	6.9	4.75	0.046	1.25 - 31.19	3.38	0.136	0.83 - 24.34
>4 beds	13	72.2	5	27.8	0.91	0.880	0.30 - 3.16	0.57	0.404	0.16 - 2.85
**Type of house**										
House without walls or that has one, two or three walls without completely enclosing a room	9	75.0	3	25.0	Ref.			Ref.		
House that has at least one room enclosed	29	65.9	15	34.1	0.64	0.552	0.13 - 2.54	0.47	0.322	0.09 - 1.95
Completely enclosed house	87	83.7	27	16.3	1.07	0.919	0.23 - 3.90	1.22	0.794	0.24 - 4.98
**Wooden wall**										
Negative	7	46.7	8	53.3	Ref.			Ref.		
Positive	118	76.1	37	23.9	3.64	0.019	1.23 - 11.06	**6.07**	**0.004**	**1.81 - 21.44**
**Wooden floor**										
Negative	73	73.0	27	27.0	Ref.					
Positive	52	74.3	18	25.7	1.07	0.852	0.54 - 2.16			
**Calamine roof**										
Negative	20	74.1	7	25.9	Ref.					
Positive	105	73.4	38	26.6	0.97	0.944	0.36 - 2.38			
**Type of water source**										
Without water or does not know	10	62.5	6	37.5	Ref.					
Potable water	5	62.5	3	37.5	1.00	1.000	0.17 - 6.33			
Well or pylon water	51	69.9	22	30.1	1.39	0.567	0.43 - 4.24			
River or rainwater	46	78.0	13	22.0	2.12	0.213	0.62 - 6.90			
Others	13	92.9	1	7.1	7.8	0.076	1.09 - 160.20			
**Fumigation**										
Negative	98	76.0	31	24.0	Ref.					
Positive	27	65.9	14	34.1	0.61	0.203	0.29 - 1.33			

OR, odds ratio, CI, confidence interval. In bold, variables with significant values (*p < 0.05). Multivariable model: Null deviance: 196.49 on 169 degrees of freedom, Residual deviance: 171.72 on 162 degrees of freedom. AIC: 187.72.

### Evolution of clinical characteristics of P. vivax symptomatic and asymptomatic infections in the Peruvian Amazon

**Figure 3:**
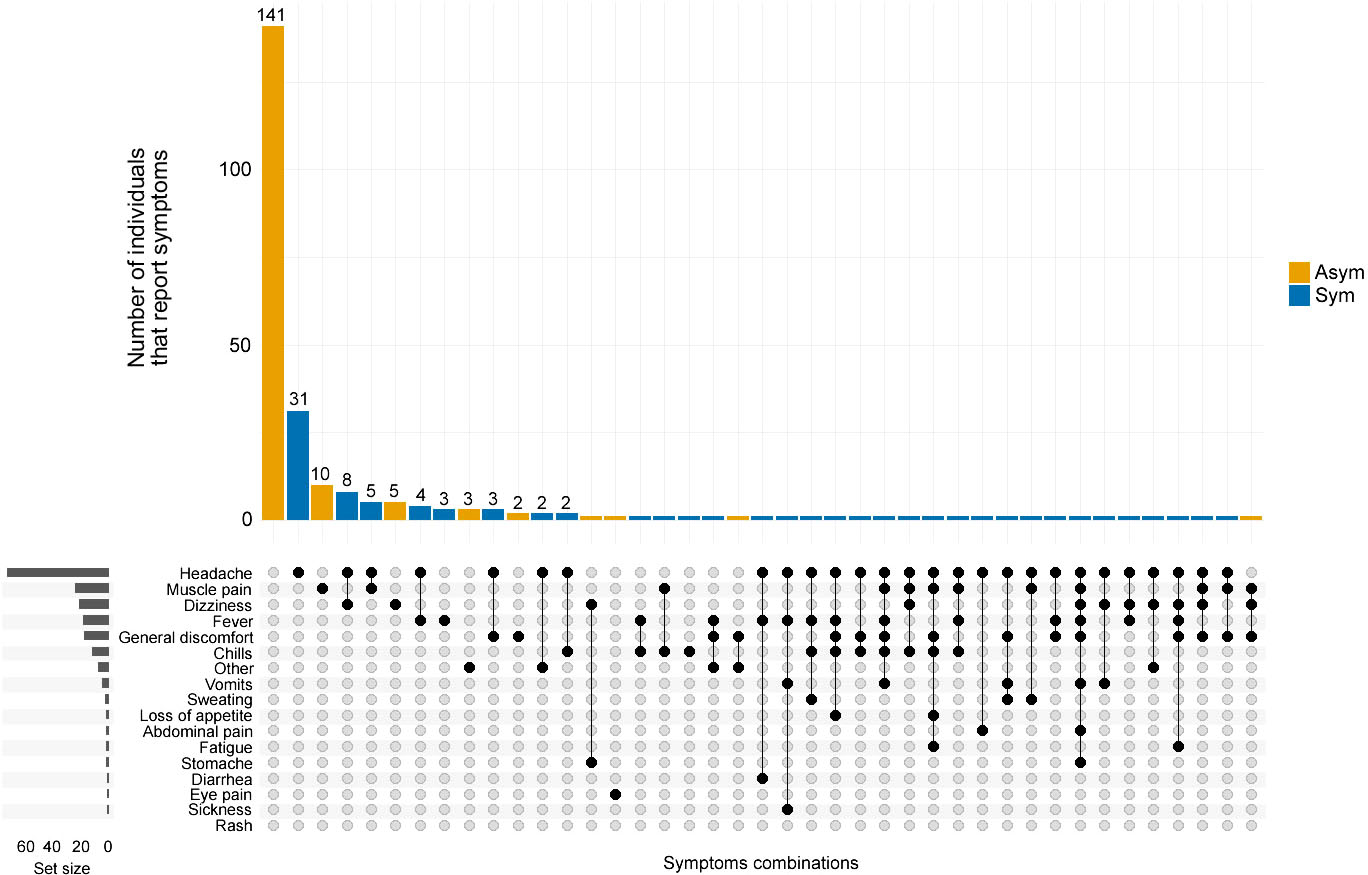
Co-occurrence of symptoms reported by *P. vivax* infected individuals. The frequency and combination of symptoms were analyzed by the clinical status of *P. vivax* infected individuals. Asymptomatic (Asym, yellow vertical bars) and Symptomatic (Sym, blue vertical bars). The horizontal grey bars to the left represent the set size of each symptom regardless of the clinical status of the infection. The intersection size represents the total of individuals that report one or a combination of symptoms pointed out by black dots below (x-axis).

From the 82 Pv Sym infections identified during the cross-sectional study, 91.5% (75/82) of the individuals experienced headaches, and only 23.2% (19/82) of individuals reported fever; 18.3% (15/82) reported dizziness, and 17.1% (14/82) reported muscle pain. Others also reported general discomfort (17.1%, 14/82) and chills (14.6%, 12/82), while some individuals also reported loss of appetite, abdominal pain, fatigue, stomach ache, diarrhea, and eye pain. Among the Pv Asym individuals, 86% (141/164) did not report any symptoms commonly associated with malaria; however, some developed symptoms including, but not limited to, muscle pain (6.7%, 11/164), dizziness (4.3%, 7/164), general discomfort (2.44%, 4/164), and eye pain (**Figure 3**).

**Figure 4:**
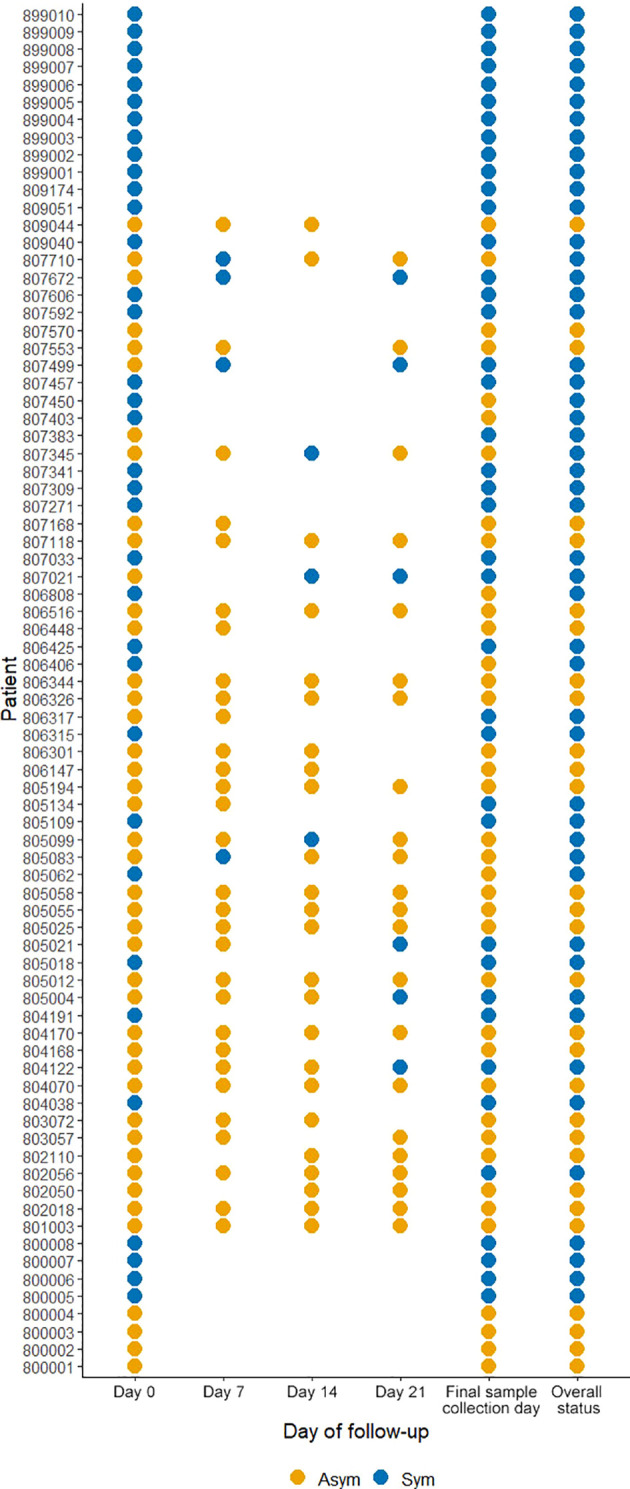
Clinical status of *P. vivax* infected individuals during follow-ups from D0 to D21. A symptomatic infection was considered whether the patients presented fever, headache, or chills. The overall status variable represents the final clinical status considering the infection status during the follow-ups and the final sample collection day. Missing dots represent missing days of follow-ups. Sym = symptomatic infections, Asym = asymptomatic infections. All dots represent a confirmed Pv infection by qPCR.

From the 246 Pv infected individuals detected by qPCR at PS (D0), only 78 individuals agreed to be followed up until D21 and at the last day of follow-up donated blood for immunological, hematological, and biochemical assays. Here, 44.9% (35/78) were classified as Pv Sym at D0, however, 17.9% (14/78) of Asym individuals classified as such at D0, experienced malaria symptoms during the follow-up weeks (28.6% (4/14) at D7, 21.4% (3/14) at D14, 21.4% (3/14) at D21), and 28.6% (4/14) on the final day of sample collection, and were, therefore, added to the symptomatic group. 6.1%, (3/49) from this group reach microscopy-detectable parasitaemia. 37% (29/78) individuals remained Asym throughout all follow-ups, within this group, five infections (17.2% (5/29)) became detectable by microscopy (40% (2/5) at D7 and 60% (3/5) at D14) (**Figure 4**). In summary, of the individuals enrolled for the case - control study, 29 remained as Asym, 49 were classified as Sym, and 30 as controls.

**Figure 5:**
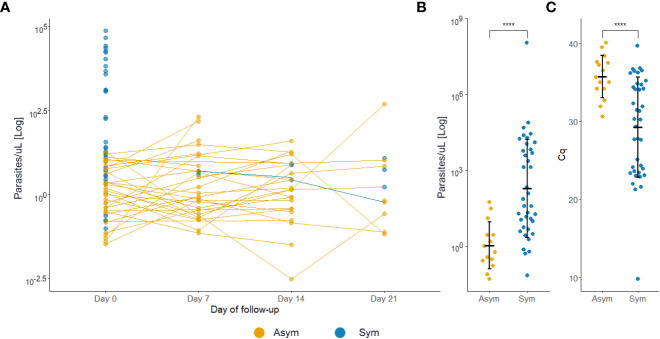
** **P*. vivax* asymptomatic infections have lower parasitemia levels compared to symptomatic patients. **(A)** Estimated parasitemia of infections during follow-ups. Each dot represents one infection. **(B)** Estimated parasitemia of infections during the final sample collection day. The parasitemia was determined by qPCR and represented by a logarithmic function. **(C)** Estimated quantification cycle (Cq) of infections during the final sample collection day. ****p<0.001, Kruskal Wallis test. Sym, symptomatic infections, Asym, asymptomatic infections.

At D0, Pv Asym infections presented a significantly lower parasitemia by qPCR (median=1.22 par/µL, IQR:0.31-5.58) compared to Pv Sym infections (median=31.49 par/µL, IQR:6.01-5268.62) (Kruskal-Wallis test, p<0.001, **Figure 5A**). Another key finding was the fluctuation of parasitemia in Pv Asym individuals during the follow-up. Further, 17.2% (5/29) of Asym Pv infections detected by qPCR were later detected by LM but remained Asym and only 6.1% (3/49) became Sym (**Figure 5A**). Individuals that remained Asym throughout follow-up (until D21) presented a significantly lower parasitemia (median=0.79 par/µL, IQR:0.27-2.74) by qPCR compared to individuals who became symptomatic (median=54.55 par/µL, IQR:6.49-6432.33) on the last day of sample collection (Kruskal-Wallis test, p<0.001, **Figure 5B, C**). Gametocytes were only detected in Pv Sym infections by microscopy (median = 1862.1 par/µL, IQR:841 - 4043.55). The lack of detection of gametocytes for Pv Asym infections occurred because these parasitemia’s were below the normal threshold of microscopy quantification. During this study, RT-qPCR was not performed for detection or quantification of mRNA from Pv gametocytes (Rovira-Vallbona et al., 2017).

### Biochemical and hematological characteristics of *P. vivax* infected individuals

**Figure 6:**
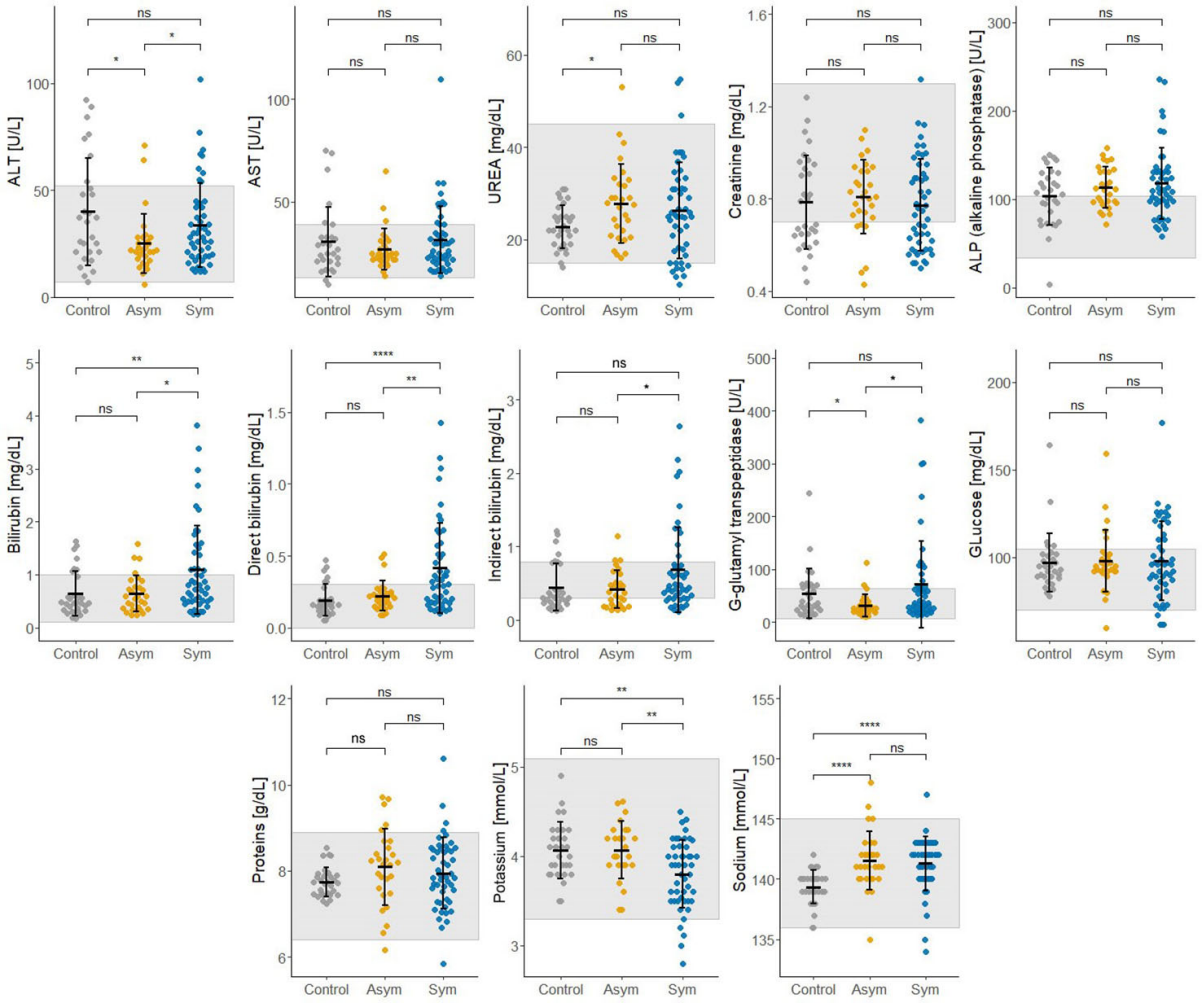
Results from the assessment of biochemical clinical analytes from enrolled individuals compared by groups (Sym/Asym/Controls). The gray area represents the normal range of expected individuals. *p<0.05, **p < 0.01, ****p < 0.0001, ns, no significant, Kruskal Wallis Test, *pos hoc*: Dunn test.

**Figure 7:**
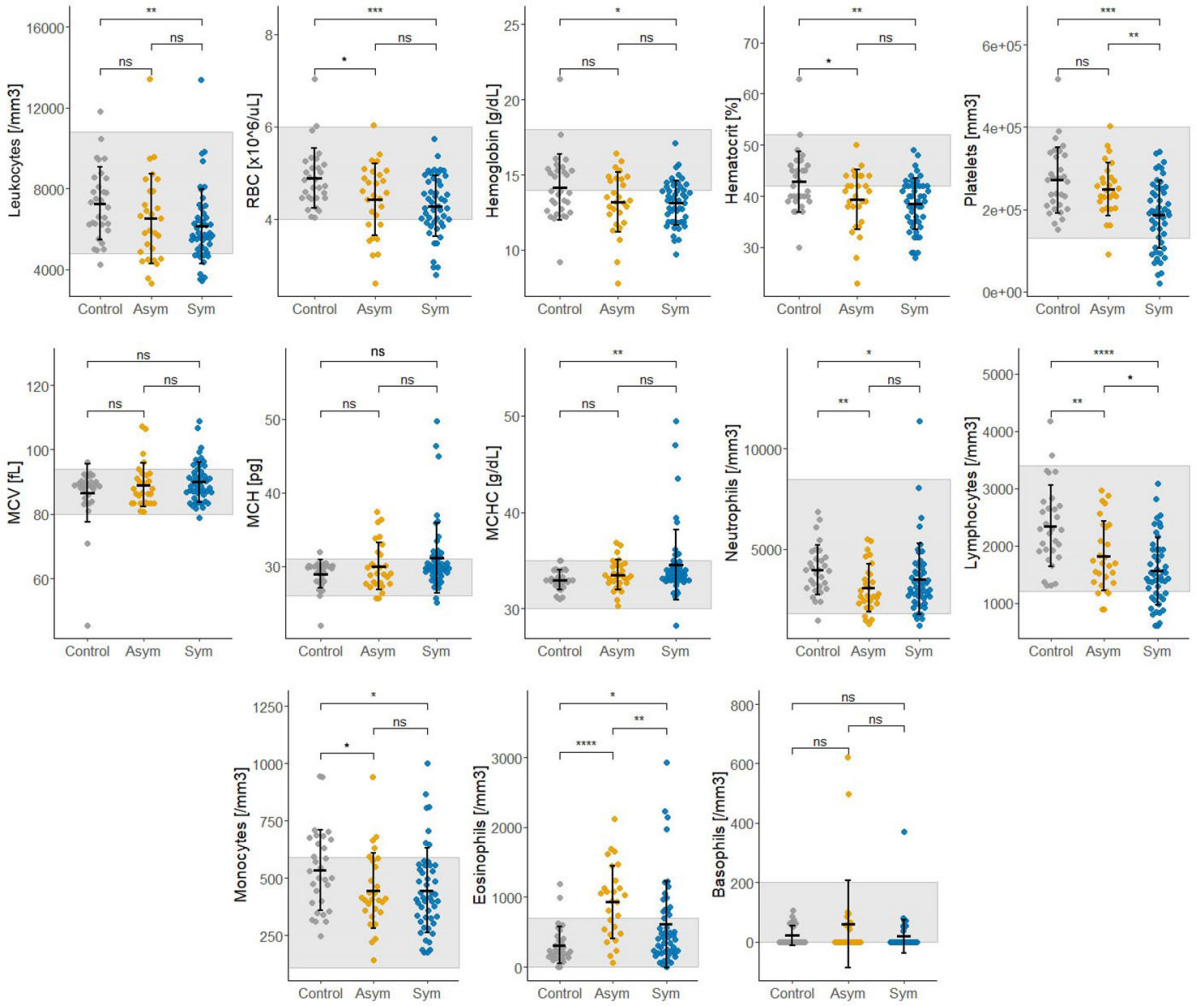
Results from the assessment of hematological clinical analytes from enrolled individuals compared by groups (Sym/Asym/Controls). The gray area represents the normal range of expected individuals. *p<0.05,** p<0.01, ***p < 0.001; ****p < 0.0001, ns, no significant, Kruskal Wallis Test, pos hoc: Dunn test.

Thirteen biochemical and thirteen hematological clinical analytes were assessed for Pv Sym, Pv Asym, and control individuals as part of the nested case - control study. Most of the median values for these parameters showed significant differences between study groups, however, the results were within the normal range, and had no clinical relevance (**Figures 6**, **7**). Instead, values for gamma-glutamyl transpeptidase hepatic enzyme and direct bilirubin were higher and outside the normal range for Pv Sym individuals in comparison to Pv Asym individuals (p<0.05) (**Figure 6**, **Supplementary Table S5**). Significantly lower values and outside the normal range were displayed by Pv Asym for hematocrit and hemoglobin clinical analytes (**Figure 7**). These values were even lower in the case of Pv Asym females in comparison to the other study groups (p<0.01) (**Supplementary Figure S4**, **Supplementary Table S5**). High eosinophil levels were observed for Pv Asym individuals compared to Pv Sym (p<0.01) and control individuals (p<0.001) (**Figure 7**, **Supplementary Table 5**).

## Discussion

Eleven population screenings were performed in thirteen communities from the Peruvian Amazon between 2018 and 2021 in search of Pv Asym individuals. The results revealed a low but heterogeneous Pv prevalence across these communities (11.4%, 95% CI [10.1 - 12.8]), where most infections were Asym (66.7%) and submicroscopic (82.9%).

The results from PS in Santa Rita, San Jose de Lupuna and San Pedro communities, located at the basin of the Nanay river, showed a median prevalence of Pv infections by qPCR of 21.3% (CI 95% [11.5 – 25.4]) and 1.25% (CI 95% [0 – 10.6]) by microscopy, where the highest prevalence was found on June-July 2019 and the lowest in January-February 2020. Notably, the mean ratios of Asym versus Sym Pv episodes during these collections showed and increment from 2019 (~3.0) to 2020 (~4.5). Furthermore, analysis of these results revealed a shift from 2018 to 2020 in Santa Rita where Pv prevalence found by qPCR only reduced by 15%, whereas Pv prevalence found by microscopy decreased by half. The assessment of Pv infections at one time point in Tarapoto and Llanchama communities, also located near to Napo River basin, and Centro Fuerte community (near to the Momon river) showed similar findings of low Pv prevalence and high proportions of Pv Asym individuals. The assessment of malaria prevalence in communities from Mazan district, such as Libertad, Primero de Enero, and Urco Miraño, along with their historical records (Carrasco-Escobar et al., 2017a; Saavedra et al., 2019; Rosas-Aguirre et al., 2021), also showed that although the number of Pv infections detected by LM had declined since 2017, qPCR detected Pv cases can be up to four times higher, and are predominantly Asym and submicroscopic. We were able to expand our study to others communities from the Mazan district such as Puerto Alegre, Huaman Urco, Salvador, and Lago Yuracyacu, where we found the same trend of results.

Due to the lack of international consensus on the definition of asymptomatic malaria (Lindblade et al., 2013), we decided to reassess the frequency and order of symptoms reported on Pv Sym and Asym individuals. One main finding from this study was that headache was the most common symptom presented in Pv Sym individuals, followed by fever, dizziness, muscle pain, general discomfort and chills. Nevertheless, the majority of studies use fever as the primary criteria for classifying malaria symptomatic individuals (Chen et al., 2021). To our knowledge, only one study from India has also reported headache as the main symptom of Pv Sym infections (Punnath et al., 2020), headache has also been described as key symptom associated to *Plasmodium ssp.* recurrences in individuals from Western Thailand (Kotepui et al., 2019). Most Pv Asym individuals did not report any symptoms at D0 or during follow-ups, but some developed symptoms such as dizziness, muscle pain, and general discomfort, which were not part of our definition criteria for Sym infections; despite those symptoms, these individuals could still perform daily activities.

Overall, Pv Asym individuals showed lower parasitemia than Pv Sym individuals by qPCR (median=0.79 par/µL vs 54.55 par/µL). Previous experiences of studies carried out by our research group in the same settings in 2015, found an estimated geometric mean of Pv parasitemia of 90 par/μL (95% CI [42–190]) where 50% of them had less than 10 par/μL (Carrasco-Escobar et al., 2017b). Age trends in infection prevalence and clinical incidence suggest an earlier acquisition of clinical immunity and more effective control of parasitemia for Pv infections compared to Pf infections (Lin et al., 2010). Pv low parasite densities can be also explained by the strict host cell preference of this species, Pv infects only reticulocytes that account for less than 1% of all erythrocytes (Gruenberg et al., 2018). However, recent work has also demonstrated the presence of a substantial proportion of the biomass of asexual Pv trophozoites and schizonts in the extravascular spaces of marrow, spleen, and liver, which could promote the condition of asymptomatic chronic malaria (Kho et al., 2021a; Kho et al., 2021b; Fernandez-Becerra et al., 2022). Ultimately, the low prevalence and parasitemia found in most study sites in this work supports the use of serology as the next step for monitoring malaria prevalence in the Peruvian Amazon as we work towards malaria elimination (Kattenberg et al., 2018).

During the case-control study, 44.9% (35/78) individuals were classified as Pv Sym at D0, 17.9% (10/78) of Asym individuals from D0, experienced malaria symptoms during the follow-up weeks and were, therefore, added to the symptomatic group (49/78), and 37% (29/78) remained Asym throughout all follow-ups. Moreover, 17.2% (5/29) of Asym infections detected by qPCR became detectable by microscopy but remained Asym and only 6.1% (3/49) became Sym at the last day of follow-up. Few longitudinal studies in Amazonia have reported data on the clinical evolution of Asym parasitemia (Branch et al., 2005; da Silva-Nunes and Ferreira, 2007; Almeida et al., 2021). Similar to our findings, a previous study by Moreno-Gutierrez et al., 2018 carried out between June–July 2015 in the Mazan district, found that from 179 qPCR Pv infected individuals, 16.8% of Pv Asym individuals experienced malaria symptoms during follow-ups but 40.8% remained Asym throughout all follow-ups, and that a small proportion (13.8%) of submicroscopic infections with positive qPCR and negative LM results became patent and detectable by LM in subsequent visits, suggesting the persistence of submicroscopic parasite carriers (Moreno-Gutierrez et al., 2018). As previously described, in this study, parasite densities in Pv Asym individuals were often low and undetectable (Imwong et al., 2016; Gruenberg et al., 2018; Nguyen et al., 2018). Further research on the clinical evolution of Asym infections is required to clarify the role of immunity in these individuals, protection against clinical malaria, and the mechanisms involved in the regulation of subclinical infections and spontaneous clearance events (Moreno et al., 2022; Roe et al., 2022).

In this study, Asym infection were prevalent during dry season. Indeed, the high prevalence of Pv Asym infections during the dry season has been frequently reported with median duration of Pv infections that can last up to 9 months, with oscillating parasitemia between ultra-low-density and high-density infections that can bridge the dry season and could become a potential source of transmission for the wet season (Nguyen et al., 2018). As Asym individuals will not seek treatment, regular population malaria-screening during dry season can aid the identification and treatment of Pv Asym individuals and therefore in the redesign of more effective malaria control and/or elimination strategies (Lindblade et al., 2013; Bhowmick et al., 2021).

Asym individuals tended to be found in houses with wooden walls, and the possession of at least one not impregnated mosquito net had a protective effect on the house, as these families had 56% decrease odds to present an Asym family member. Our results are similar from a study conducted in 2009 in individuals that lived in the forest of central Vietnam, with resembling living conditions as the participants from our study (Thanh et al., 2015). In malaria endemic areas of the forest of central Vietnam, it is common to found wooden houses that had been built by the government, not reflecting the actual socio-economic status of respective dwellers (Thanh et al., 2015). In our study sites, houses are mainly made from wooden walls, tin ceiling and have soil floors, reflecting the low socio-economic status of their inhabitants, however, wooden walls was the only house material associated to Asym malaria infection. Interestingly, even having a mosquito net without insecticide per house showed a protective effect from having an Asym family member, highlighting again the proper use of mosquito nets as a protective measure against malaria infection (Thanh et al., 2015). Current studies, carry out by our research group, are exploring more in detail the socio-demographic characteristics, and behavior or habits of these individuals in the search of other malaria associated factors.

Importantly, we explored the clinical characteristics of Pv infected individuals by comparing the biochemical and hematological parameters between Pv Asym and Sym. Several studies had previously reported different levels of hematological and biochemical parameters in Pv acute infections, but most of these parameters returned to normal values during convalescence or following antimalarial treatment (Kotepui et al., 2015; O’Connell and Nutman, 2015; Tobón-Castaño et al., 2015; Awoke and Arota, 2019; Gupta et al., 2019; Alves-Junior et al., 2020; Ullah et al., 2020). Individuals with Asym malaria had shown low platelet count (<150 x 10^9^/L) as well increased levels of Protein Reactive C (Igbeneghu et al., 2011; de Mast et al., 2015). Recently, Almeida et al. (2021) found no relevant alterations for hematological and biochemical parameters between Pv Sym and Pv Asym vs control individuals from Brazil, except for signs of lymphopenia and thrombocytopenia along with high levels of Protein Reactive C, as well as lower levels of cholesterol in Pv Sym individuals (Almeida et al., 2021). In contrast, values such as platelets (≥185,000 cells/μL), Protein Reactive C (≤2 mg/dL), and total bilirubin (≤0.28 mg/dL) had been suggested as an adjunctive tool to estimate the probability of having a blood film negative for malaria, but with confirmation by a negative thick and thin blood films by LM (Leli et al., 2020)

This research is the first case - control study that evaluates hematological and biochemical laboratory indicators in Pv Sym and Asym infected individuals from the Peruvian Amazon versus controls. One of the key findings was that Pv Asym individuals had low values for hematocrit and hemoglobin, and these values were even lower in Pv Asym women (media Hb =11,25 g/dL; parasitemia: median=1.54 par/µL), categorizing them with mild anemia (World Health Organization, 2011), however, we cannot conclusively demonstrate this due to low sample size (n=4). The presence of anemia in adult women with Pf submicroscopic infection has been previously reported (Pava et al., 2016; Woolley et al., 2021), however, this remains to be investigated for Pv submicroscopic and Asym infections. Interestingly, high levels of eosinophils found in Pv Asym and Sym individuals from this study could be associated with self-reports of the presence of intestinal parasites in 35% and 53% of Pv Asym and Sym individuals (Sánchez-Arcila et al., 2015). Values for gamma-glutamyl transpeptidase hepatic enzyme and bilirubin were higher and outside the normal range for Pv Sym individuals compared to Pv Asym individuals (p<0.05). Elevations in γGT and ALP have also been reported in relation to liver injury in uncomplicated malaria (Reuling et al., 2018). Moreover, bilirubinemia has been suggested as a common marker of Pv acute infection and as a predictor of infection severity (Alves-Junior et al., 2020; Odedra et al., 2020; Almeida et al., 2021).

Additional results from physical evaluations of the participants showed normal corporal mass index and no palpable livers or spleens were reported (data not shown). This finding reinforces results from a previous study that Pv Asym infections could go unnoticed by laboratory routine exams or clinical evaluation (Almeida et al., 2021). We argue that hematological and biochemical clinical parameters outside the normal range in Pv infected individuals are most likely attributable to specific living conditions in these endemic areas, nutritional status or other no reported comorbidities by the participants because they were unaware of it. Future studies will associate results from hematological and biochemical clinical parameters with innate and adaptive immune response biomarkers to Pv infection. The generation of more data about hematological and biochemical laboratory parameters in Asym malaria infection will be useful for its incorporation into clinical decision-support algorithms for its diagnosis, monitoring response to treatment, and differentiation from other acute febrile illnesses in these endemic settings (Morang’a et al., 2020).

One of the main limitations of this study was the absence of other pathogen screenings, the exploration of micronutrient deficiencies and the immunological and genetic backgrounds of the participants. This information was only registered by interview and could have been fundamental for the interpretation of hematological and biochemical clinical parameters between Pv Sym and Asym individuals. Futures studies will integrate these epidemiological data with results of immunological studies on PBMCs, plasma, and serum samples from the same individuals in order to better characterized the innate and acquired immunological response of Pv Asym individuals in the Peruvian Amazon. This information is relevant to augment the general knowledge of asymptomatic condition in infectious diseases.

## Conclusions

In this study, we compared different epidemiological, clinical and biochemical characteristics, as well as hematological parameters between Sym and Asym Pv infected individuals, and control individuals from the Peruvian Amazon, in the search of biomarkers of clinical immunity to Pv infection. Our data revealed a low but heterogeneous Pv prevalence across communities, where most infections were Asym and submicroscopic. During this study, most Sym individuals experienced headache, and not fever, as a common symptom. As previously reported, we observed fluctuation of low parasitemia in Pv Asym individuals during the follow-ups and only a small proportion of these individuals became Sym or microscopy detectable. Further, we identified that houses with wooden walls were associated to Asym infections and that unimpregnated mosquito nets had a protective effect against Asym infections. Finally, we showed that Pv Asym infections may go unnoticed by laboratory routine exams or clinical evaluation. A solid understanding of malaria Asym infections is important for the design of novel malaria control strategies as the country works towards to its elimination.

## Data availability statement

The original contributions presented in the study are included in the article/**Supplementary Material**. Further inquiries can be directed to the corresponding author.

## Ethics statement

The studies involving human participants were reviewed and approved by Ethical Committee of the Universidad Peruana Cayetano Heredia (Lima, Peru). The patients/participants provided their written informed consent to participate in this study.

## Author contributions

KT, DG, JV, and EV contributed to the conception and design of the study. EV, KG, JT, KT, AC, MG, and CR performed the methodology. SSGC, JG and CA performed the statistical analyses. Investigation: EV, SG, and KT. Resources: KT, DG JMV, and EV. Data Curation: SSGC, MG, EV, KT, JT, and JG. Writing – original draft preparation: EV, SSGC, JG, and KT. Writing – review and editing: EV, SSGC, JG, KG, JT, KT, AC, MG, CR, CA, DG, and JV. Visualization: SSGC, JG, and CA. Supervision: KT, DG, and JV. Project administration: KT, DG, and JV. Funding acquisition: KT, EV, DG, and JV. All authors contributed to manuscript revision and approved the submitted version.

## Funding

Funding for this study was provided by The International Centers of Excellence for Malaria Research (ICEMR) program (U19AI089681), NIH–USA; FOGARTY International Center (D43TW007120), NIH-USA. EV is supported by Proyecto Concytec – Banco Mundial “Mejoramiento y Ampliación de los Servicios del Sistema Nacional de Ciencia Tecnología e Innovación Tecnológica” 8682-PE, through its executing unit ProCiencia [08-2018-FONDECYT/BM-Programas de Doctorados en Áreas Estratégicas y Generales].

## Acknowledgments

We thank all residents and local authorities from the communities for their enthusiastic participation in the study, as well as all field workers for their dedication during the fieldwork. EV acknowledges the financial support of Proyecto Concytec – Banco Mundial “Mejoramiento y Ampliación de los Servicios del Sistema Nacional de Ciencia Tecnología e Innovación Tecnológica” 8682-PE, through its executing unit ProCiencia [08-2018-FONDECYT/BM-Programas de Doctorados en Áreas Estratégicas y Generales]. We thank Oscar Nolasco and Roberson Ramirez for their technical support for during qPCR experiments. We also thank logistic and administrative support of Rosa Alban, Yulissa Vasquez, and Yovana Maldonado from the Malaria Laboratory at UPCH.

## Conflict of interest

This study received funding from the World Bank Group. The funder was not involved in the study design, collection, analysis, interpretation of data, the writing of this article or the decision to submit it for publication.

The authors declare that the research was conducted in the absence of any commercial or financial relationships that could be construed as a potential conflict of interest.

## Publisher’s note

All claims expressed in this article are solely those of the authors and do not necessarily represent those of their affiliated organizations, or those of the publisher, the editors and the reviewers. Any product that may be evaluated in this article, or claim that may be made by its manufacturer, is not guaranteed or endorsed by the publisher.

## Supplementary material

Supplementary Figure 1 Hotspots of *P. vivax* asymptomatic cases in communities with multiple population screenings (PS) throughout the study. Santa Rita (SR), San José de Lupuna (SJL) and Urcomiraño (UM).

Supplementary Figure 2 Hotspots of *P. vivax* asymptomatic cases in communities with one population screenings (PS). Primero de Enero (PE), Huaman Urco (HU), Lago Yurac Yacu (LYY), and Centro Fuerte (CF).

Supplementary Figure 3 Hotspots of *P. vivax* asymptomatic cases in Libertad community.

Supplementary Figure 4 Results from hemoglobin, hematocrit and creatinine analyzed by sex **(A)** Male **(B)** Female. The gray area represents the normal range of expected individuals. *p<0.05, ***p < 0.001; ****p < 0.0001, ns no significant, Kruskal Wallis Test, pos hoc: Dunn test.
