# Supplementary figures and images for: Epidemiological characteristics of P. vivax asymptomatic infections in the Peruvian Amazon
Source: Front Cell Infect Microbiol. 2022 Aug 31;12:901423. doi: 10.3389/fcimb.2022.901423 (PMC9471197; doi:10.3389/fcimb.2022.901423)

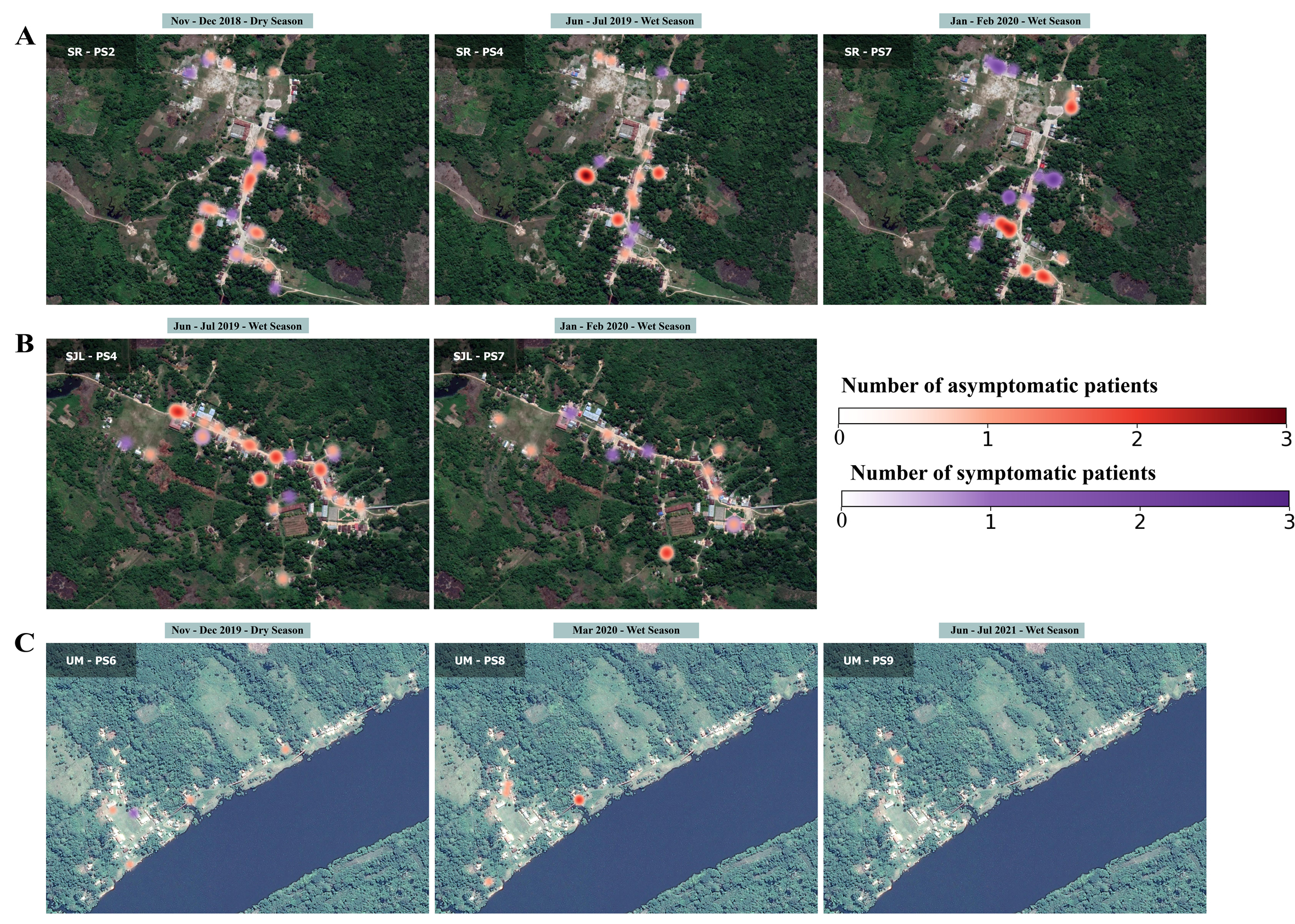

Supplement: Supplementary Figure 1 — Hotspots of P. vivax asymptomatic cases in communities with multiple population screenings (PS) throughout the study. Santa Rita (SR), San José de Lupuna (SJL) and Urcomiraño (UM). [file Image_1.jpeg]

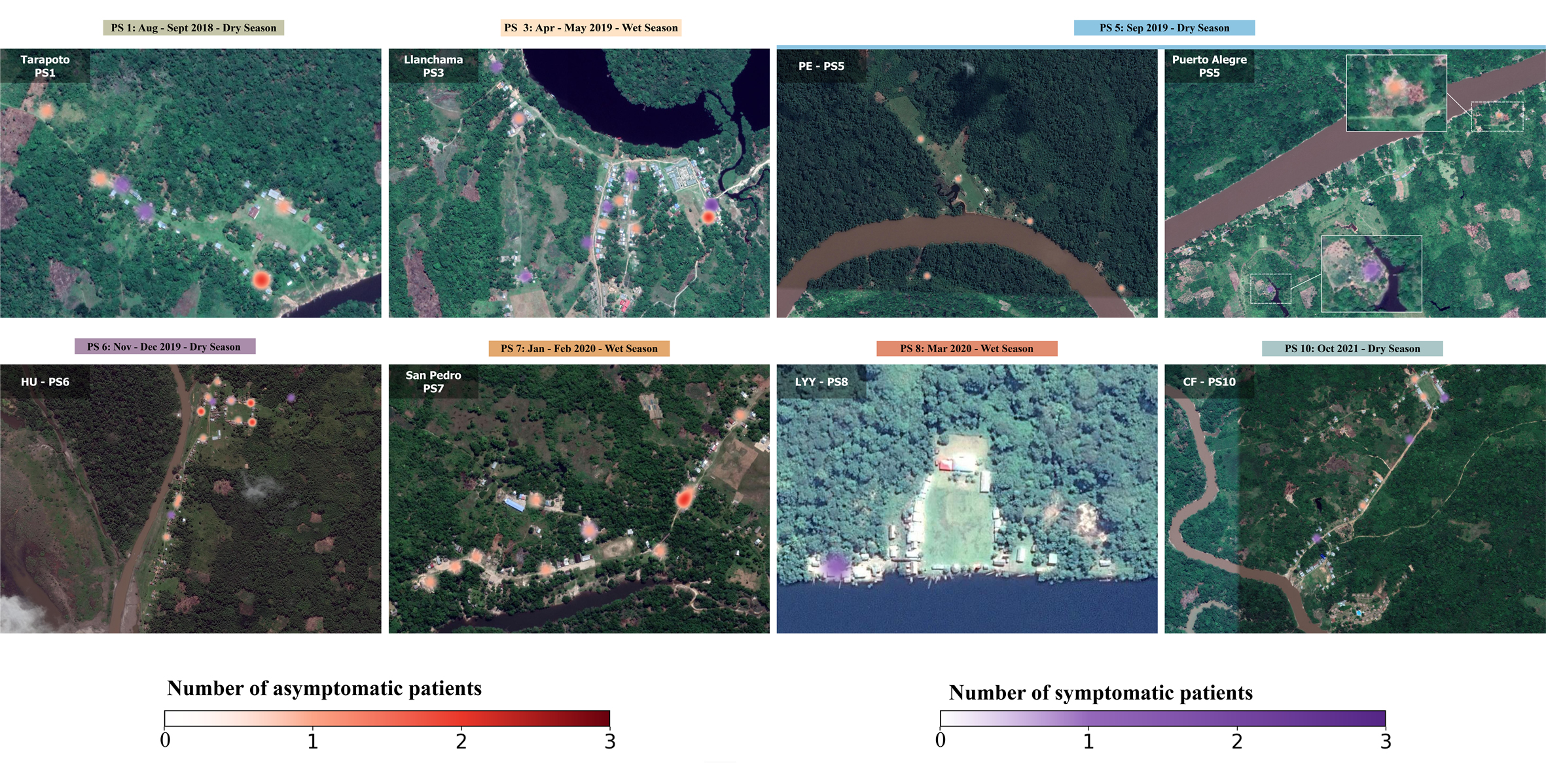

Supplement: Supplementary Figure 2 — Hotspots of P. vivax asymptomatic cases in communities with one population screenings (PS). Primero de Enero (PE), Huaman Urco (HU), Lago Yurac Yacu (LYY), and Centro Fuerte (CF). [file Image_2.jpeg]

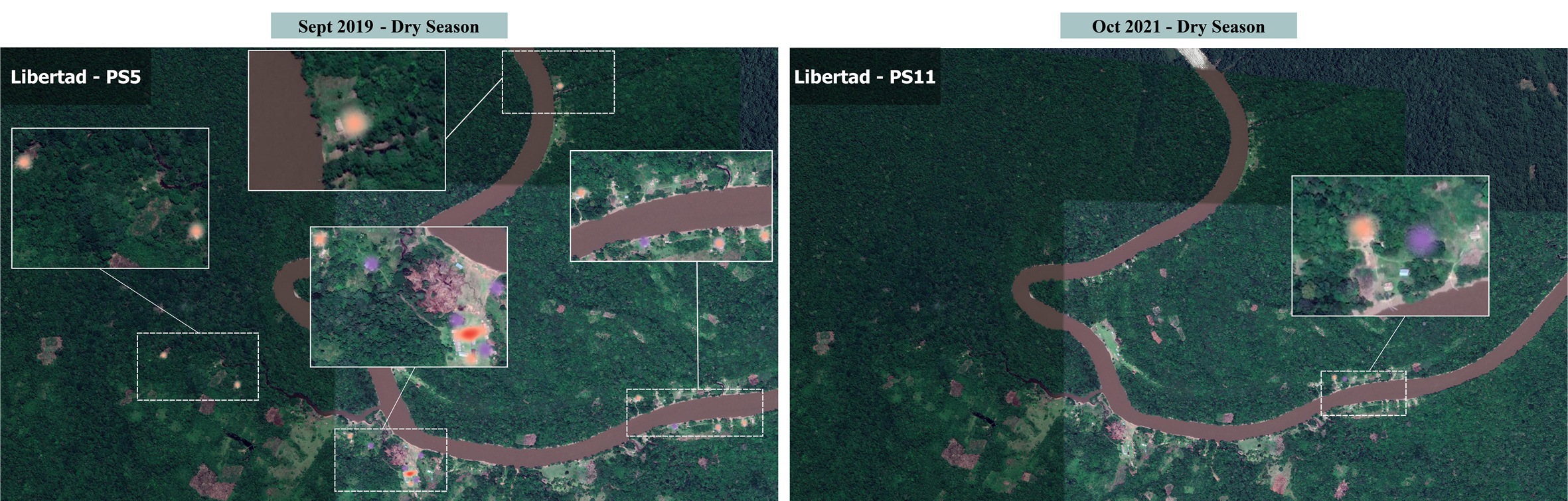

Supplement: Supplementary Figure 3 — Hotspots of P. vivax asymptomatic cases in Libertad community. [file Image_3.jpeg]

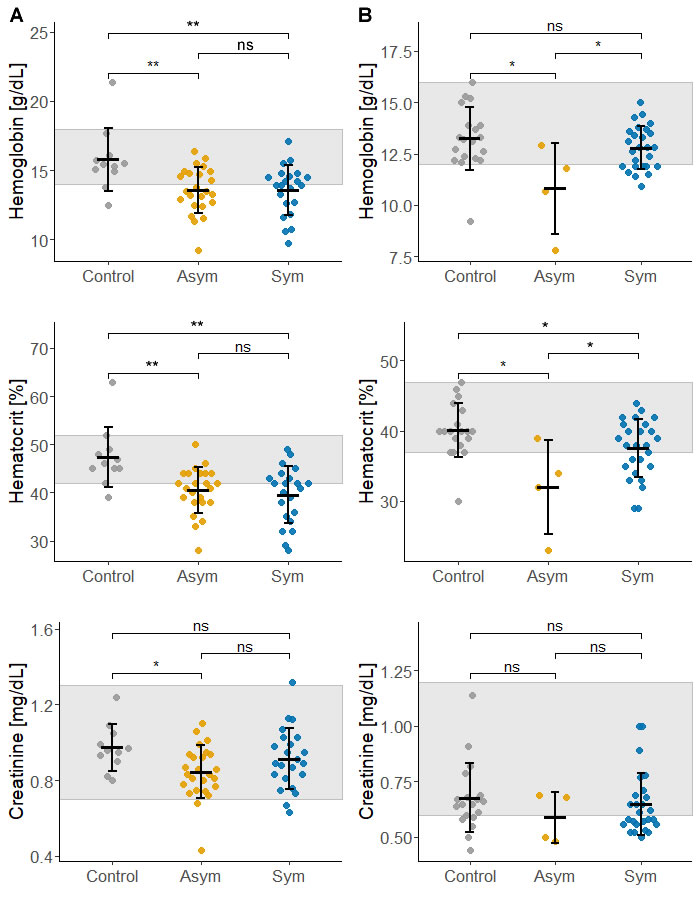

Supplement: Supplementary Figure 4 — Results from hemoglobin, hematocrit and creatinine analyzed by sex (A) Male (B) Female. The gray area represents the normal range of expected individuals. *p<0.05, ***p < 0.001; ****p < 0.0001, ns no significant, Kruskal Wallis Test, pos hoc: Dunn test. [file Image_4.jpeg]
