# Supplementary material for: Epidemiological characteristics of P. vivax asymptomatic infections in the Peruvian Amazon
Source: Front Cell Infect Microbiol. 2022 Aug 31;12:901423. doi: 10.3389/fcimb.2022.901423 (PMC9471197; doi:10.3389/fcimb.2022.901423)
Supplement: Supplementary file 6 [file Table_2.docx]

**Supplementary Figures captions**

**Supplementary Figure 1.** Hotspots of *P. vivax* asymptomatic cases in communities with multiple population screenings (PS) throughout the study. Santa Rita (SR), San José de Lupuna (SJL) and Urcomiraño (UM)

**Supplementary Figure 2.** Hotspots of *P. vivax* asymptomatic cases in communities with one population screenings (PS). Primero de Enero (PE), Huaman Urco (HU) Lago Yurac Yacu (LYY) and Centro Fuerte (CF)

**Supplementary Figure 3**. Hotspots of *P. vivax* asymptomatic cases in Libertad community

**Supplementary Figure 4**. Results from Hemoglobin, Hematocrit and Creatinine analyzed by sex (A) Male (B) Female. The gray area represents the normal range of expected individuals. *p<0.05, ***p < 0.001; ****p < 0.0001, ns no significant, Kruskal Wallis Test, pos hoc: Dunn test.
